# Editorial: Effects of hormonal contraceptives on the brain

**DOI:** 10.3389/fendo.2023.1129203

**Published:** 2023-01-31

**Authors:** Belinda Pletzer, Erika Comasco, Esmeralda Hidalgo-Lopez, Agnès Lacreuse, Birgit Derntl

**Affiliations:** ^1^ Department of Psychology & Centre for Cognitive Neuroscience, Paris-Lodron-University Salzburg, Salzburg, Austria; ^2^ Department of Women´s and Children´s Health, Science for Life Laboratory, Uppsala University, Uppsala, Sweden; ^3^ Department of Psychological and Brain Sciences, University of Massachusetts, Amherst, MA, United States; ^4^ Department of Psychiatry and Psychotherapy, Tübingen Center for Mental Health (TüCMH), University of Tübingen, Tübingen, Germany

**Keywords:** hormonal contraceptives, progestins, intra-uterine device (IUD), brain, behavior

Hormonal contraceptives just celebrated their 60th anniversary and are used by 150 million women worldwide (1). While the brain, as a neuroendocrine organ, is in fact the primary target of hormonal contraceptives, we know relatively little about their effects on the brain beyond the suppression of the hypothalamic-pituitary-gonadal axis (2). Combined oral contraceptives (COCs), as the most commonly used form of hormonal contraceptives, downregulate endogenous hormone production, abolish the cyclical fluctuations of endogenous hormones and replace them by constant levels of potent synthetic estrogens and progestins (3). These mechanisms may affect brain areas with a high sensitivity to estrogenic and progestagenic actions (for reviews see 4, 5), as has been demonstrated for the hippocampus (6, 7), amygdala (8–10) and prefrontal cortex (8, 11, 12) among others. These areas are involved in cognitive and emotional processing, and there is increasing awareness of potential alterations in these processes due to hormonal contraceptive use (13).

In fact, first evidence indicating an association between the use of COC and depressive symptoms dates back to the 1960s (14). Nowadays, several studies have shown that oral contraceptive use may result in improved and stabilized mood in some women (15), but mood worsening and depressive symptoms in others (16). Some studies see a blunted cortisol response as partly responsible for the altered vulnerability of COC users to mood disorders (17), an effect that was also highlighted in the current Research Topic (Hogsted et al.). Yet, it is still unclear, which factors determine how a woman will react to COC treatment. Understanding the mechanisms underlying this dissociation of two groups of women with differential affective responsivity to COC treatment is critical, from a medical as well as a methodological viewpoint. Adverse mood effects are among the most common reasons for COC discontinuation (as also pointed out in this topic by Pletzer et al.) thus introducing substantial sampling bias in studies on long-term COC users. It has been suggested that adolescent onset of COC use, as well as previous depressive episodes may represent risk factors for developing adverse mood symptoms during COC treatment (18). In contrast, it has been discussed, whether particularly women with premenstrual dysphoric disorder (PMDD) profit from the mood-stabilizing effects of COCs. Indeed, some COCs with anti-androgen activity have been approved for the treatment of premenstrual dysphoric disorder (19), though recent meta-analyses suggest that in comparison with placebo women with PMDD show no significant improvement in terms of depressive symptoms upon treatment with COCs (20).

In the current Research Topic, seven articles address the complex interactions between COC use and emotional processing in different female samples (Beinhölzl et al.; Doornweerd et al.; Larsen et al.; Lewis et al.; Pletzer et al.; Robertson et al.; Schmidt et al.).

Focusing on the risk factor of adolescent onset, Doornweerd et al. followed depression and anxiety trajectories of adolescent girls between the age of 13 until 18. Their results demonstrate that girls who never used COCs during adolescence showed increased levels of depressive and anxiety symptoms in late adolescence compared to girls who started using COCs at some point during the study. These results are in line with the several studies indicating potential mood-stabilizing effects of OC intake, particularly in women who do not develop adverse mood symptoms during the first months of treatment and thus become long-term COC users (21), but are in contrast to studies suggesting that adolescent onset of COC use is associated with higher risk for mood disorders (18, 22). However, COC users showed higher risky behavior and also differed in several aspects from never users. This study indicates that COC use needs to be considered in the development of internalizing symptoms from adolescence into adulthood, nicely points out that relevant confounders need to be considered, and adds to research showing that COC use is associated with mental health.

Given that a history of depression represents a risk factor for developing depressive symptoms during COC use (18), depressed patients are of particular interest with regards to their neuro-behavioral responses to COC use. In the current Research Topic, two manuscripts focused on a population with depression and evaluated the efficacy of antidepressant treatment in COC users at the behavioural (Beinhölzl et al.) and neurotransmitter level (Larsen et al.). They found no strong evidence for a difference in treatment response between COC users and non-users diagnosed with depression (Larsen et al.), but also no evidence for changes in emotional attention during a one-week anti-depressant treatment in healthy COC users (Beinhölzl et al.).

Focusing on women diagnosed with PMDD, Robertson et al. investigated the feasibility of a modern COC pill containing 17-beta estradiol rather than ethinylestradiol as an estrogenic component and the new anti-androgenic progestin nomegestrol acetate. The results demonstrated that 20% of women reported emotional side effects and discontinued COC treatment, but the majority of women (75%) reported positive mood changes and reduced depression, anxiety and stress scores during COC treatment.

While the majority of previous studies on emotional processing in COC users have focused on negative emotionality using aversive stimulus material, Schmidt et al. investigated the neural correlates of positive stimulus processing in COC users. However, results revealed no significant group or phase differences in either subjective stimulus evaluations or neural reactivity towards positive stimuli. Null findings were also obtained with regards to value-based decision making during COC use in comparison to naturally cycling females (Lewis et al.) and associations between personality factors and contraceptive choice (Pletzer et al.). The systematic reporting of null findings supported by Bayesian analyses is vital in avoiding publication bias and providing a balanced overview of the aspects of human emotion and cognition affected by hormonal contraceptive use.

Regarding cognitive changes during hormonal contraceptive use, previous studies have yielded mostly inconsistent results due to mostly small sample sizes and a lack of control for the contraceptive formulation used (23). A systematic review arrived at the conclusion that the most consistent finding is a moderate increase in memory tasks, while results regarding spatial performance are inconclusive, but may depend on the hormonal contraceptive formulation used (24). In this Research Topic, three articles have addressed the issue of spatial abilities during COC use including well-powered samples and strictly controlling for both, the progestin type and estrogen dose contained in the COC (Hampson et al.; Koebele et al.; Noachtar et al.).

While previous studies suggested that mostly the androgenicity of the progestin determined the directionality of COC dependent effects on spatial abilities, Hampson et al. observed only moderate effects of androgenicity, while estrogenic potency had a more substantial impact. Supporting the idea of a role for ethinylestradiol in diminishing spatial performance, Koebele et al. observed beneficial effects of the anti-androgenic progestin drospirenone on spatial working memory in rats, which were reversed by ethinylestradiol. Furthermore, ethinylestradiol increased the expression of glutamate decarboxylase (GAD), hinting at inhibitory effects, in the perirhinal cortex. Also, in the study by Noachtar et al., deactivation of the caudate and postcentral gyrus during a spatial navigation task was more prominently related to the duration of COC use in users of levonorgestrel containing pills. Connectivity patterns however were related to the duration of COC use irrespective of androgenicity. Thus, both human studies suggest only moderate roles of androgenicity in the modulation of spatial abilities by COC at the behavioral (Hampson et al.) and brain levels (Noachtar et al.). The studies do however report contrary findings regarding verbal fluency performance: While Hampson et al. observed moderate increases in the number of words produced during the active intake phase, Noachtar et al. found a negative association between verbal fluency performance and longer duration of COC use. The results suggest potentially differential effects of short- and long-term contraceptive use on verbal memory.

Importantly, all three studies go well beyond the traditional cross-sectional design, comparing COC users to naturally cycling women, but rather rely on longitudinal and correlational designs focusing on short term hormone administration, withdrawal or the duration of COC use. An even more stringent longitudinal approach was chosen by Jensen et al., who followed a single subject’s resting brain connectivity over two whole cycles – one with and one without COC treatment. Modularity, system segregation and characteristic path length were significantly higher across the natural compared to the contraceptive cycle, hinting at alterations in the hierarchical organization of resting brain networks during COC use. Together with a previous study using the same approach (25), these network analyses particularly highlight the effects of an absent cyclicity of hormonal fluctuations in COC users, rather than focusing on the reduction in endogenous hormone levels or the potency of the synthetic hormones administered. However, in the majority of studies, it cannot be determined, which of the neuroendocrine mechanisms described above are responsible for the changes observed during COC treatment. In that respect, levonorgestrel containing intra-uterine devices (IUDs) may represent a new frontier for the neuroscience of hormonal contraception given that hormonal IUDs do not abolish endogenous hormonal fluctuations (for review see 26). First neuroimaging data from IUD users are presented within this Research Topic by Beltz et al.. IUD users provide a promising natural experiment for the interplay between exogenous and endogenous sex hormones, and they are likely qualitatively different from OC users with whom they are often grouped in hormonal contraceptive research.

Finally, with respect to delineating the mechanisms underlying hormonal contraceptive-dependent changes in the brain, this Research Topic particularly benefits from the integration of human neuroimaging studies with animal research shedding light on the mechanisms at work (Koebele et al.; Huang et al.). The benefits of this inter-disciplinary integration are manifold. While human neuroimaging studies may identify regions of interest for microstructural analyses in animals, animal studies are for example able to delineate the effects of the estrogenic and progestagenic components of COCs, as outlined in the work by Koebele et al. within this topic. These approaches may in turn lay the groundwork for future neuroimaging studies in humans.

In summary, 14 manuscripts addressing a variety of topics in the realm of hormonal contraception are enclosed in this special issue. We are particularly proud to point out that the majority of first and senior authors on these articles are female. This collection of papers offers a wide range of approaches/perspectives to help our understanding of synthetic hormones’ effects and to open new paths in this field of research. Particularly, these studies increase our understanding of temporal relationships through longitudinal studies, as well as hormonal contraceptive type-specific effects on brain, behaviour and mental health. With neuroendocrinological research thriving in the past decades, it now seems within our grasp to deepen our understanding of the complex relationship between synthetic hormones, cognition and emotional well-being. After 60 years of hormonal contraceptive use, it seems like high time to do so.

## Author contributions

BP wrote the first draft of the manuscript. EC, EH-L, AL and BD provided revisions, which were incorporated by BD and BP. All authors have read and approved the final manuscript.
